# Predicting suicide risk in 137,112 people with severe mental illness in Finland: external validation of the Oxford Mental Illness and Suicide tool (OxMIS)

**DOI:** 10.1038/s41398-023-02422-5

**Published:** 2023-04-18

**Authors:** Amir Sariaslan, Thomas Fanshawe, Joonas Pitkänen, Andrea Cipriani, Pekka Martikainen, Seena Fazel

**Affiliations:** 1 Department of Psychiatry, University of Oxford, Warneford Hospital, Oxford, UK; 2 Nuffield Department of Primary Care Health Sciences, University of Oxford, Oxford, UK; 3 Population Research Unit, Faculty of Social Sciences, University of Helsinki, Helsinki, Finland; 4 Centre for Health Equity Studies (CHESS), Stockholm University and Karolinska Institutet, Stockholm, Sweden; 5 Max Planck Institute for Demographic Research, Rostock, Germany

**Keywords:** Schizophrenia, Bipolar disorder

## Abstract

Oxford Mental Illness and Suicide tool (OxMIS) is a standardised, scalable, and transparent instrument for suicide risk assessment in people with severe mental illness (SMI) based on 17 sociodemographic, criminal history, familial, and clinical risk factors. However, alongside most prediction models in psychiatry, external validations are currently lacking. We utilised a Finnish population sample of all persons diagnosed by mental health services with SMI (schizophrenia-spectrum and bipolar disorders) between 1996 and 2017 (*n* = 137,112). To evaluate the performance of OxMIS, we initially calculated the predicted 12-month suicide risk for each individual by weighting risk factors by effect sizes reported in the original OxMIS prediction model and converted to a probability. This probability was then used to assess the discrimination and calibration of the OxMIS model in this external sample. Within a year of assessment, 1.1% of people with SMI (*n* = 1475) had died by suicide. The overall discrimination of the tool was good, with an area under the curve of 0.70 (95% confidence interval: 0.69–0.71). The model initially overestimated suicide risks in those with elevated predicted risks of >5% over 12 months (Harrell’s E_max_ = 0.114), which applied to 1.3% (*n* = 1780) of the cohort. However, when we used a 5% maximum predicted suicide risk threshold as is recommended clinically, the calibration was excellent (ICI = 0.002; E_max_ = 0.005). Validating clinical prediction tools using routinely collected data can address research gaps in prediction psychiatry and is a necessary step to translating such models into clinical practice.

## Introduction

Prognostic models and tools have been increasingly implemented in routine clinical practice and support clinical decision-making in other areas of medicine, including cardiovascular [1] and cancer medicine [2–4]. However, their use is inconsistent and highly variable in mental health. Part of the reason for this is that prediction models in mental health are very rarely externally validated [5]. Such validation is a necessary step on the path to implementation alongside work on feasibility, acceptability and clinical impact. In the suicide field, this is not different—few models have been externally validated despite their clinical use in some settings [6]. Clinical surveys have found that despite routine use of risk tools in most emergency departments, around two-thirds relied on unvalidated and locally-developed instruments [7]. Where external validations have been reported for suicide risk assessment tools, they have commonly omitted key performance measures [8], particularly calibration, which tests the agreement between observed and predicted outcomes, that is necessary to appraise them.

Oxford Mental Illness and Suicide tool (OxMIS) is a brief, scalable, structured risk assessment tool designed to assess the risk of suicide in patients with severe mental illness (schizophrenia-spectrum disorders or bipolar disorder). The tool was developed and validated in a cohort of 75,158 individuals diagnosed with severe mental illness in Sweden, is available as a free web-based calculator with its coefficients published (https://oxrisk.com/oxmis) and protocol available [9]. The model provides probability scores for a 12-month suicide risk for individuals with SMI (with a ceiling of 5%) with good measures of discrimination (AUC = 0.71), and calibration. As OxMIS has not been validated in a new independent sample, we aimed to test its performance using population-based data from Finland over a 22-year period, which allowed us to examine the predictive performance of the tool in over 137,000 persons with SMI.

## Subjects and methods

### Data

A unique civic registration number is assigned to every resident of Finland, allowing for the precise linking of multiple administrative population registers [10]. Following approvals by the Ethics Board of Statistics Finland (TK-53-1490-18) and the Finnish Institute for Health and Welfare (THL/2180/14.02.00/2020), we were granted access to pseudonymized data from governmental agencies in FIONA, which is a secure remote access platform maintained by Statistics Finland. In Finland, informed consent is not required for population-based register studies of this nature. The lead author of the study (AS) generated the final dataset, which included all predictors and the outcome measure, and performed all statistical analyses on the FIONA platform.

We initially used the Care Register for Health Care [11], which is maintained by the Finnish Institute for Health and Welfare, to identify all individuals between the ages of 15 and 65 years who were diagnosed with a severe mental illness between 1 January 1996 and 31 December 2017. The Care Register for Health Care includes all inpatient hospital episodes in Finland since 1 January 1970, classified according to the International Classification of Diseases (ICD; eighth revision [ICD-8]: 1970–1986; ninth revision [ICD-9]: 1987–1995; tenth revision [ICD-10]: 1996–2017). The register also includes outpatient specialist visits in secondary care using the same classification system, albeit during a more limited period (1998–2017) compared to the inpatient care data. Data on treatments provided during care were not available. Severe mental illness (SMI) was defined as an episode of either schizophrenia-spectrum disorder (ICD-8: 295, 297–298; ICD-9: 295, 297–299; ICD-10: F20-F29) or bipolar disorder (ICD-8: 296 [excluding 296.2]; ICD-9: 296 [excluding 296.1A-D, 296.1F-G], ICD-10: F30-F31). We used a hierarchical definition, where individuals who had been diagnosed with both a schizophrenia-spectrum disorder and bipolar disorder were considered to have the former. External validation studies of single-episode diagnoses of schizophrenia-spectrum disorders (positive predictive value, PPV ≥ 84%) [12], bipolar disorder (PPV range between 87–93%) [13] have demonstrated excellent validity.

Using this approach, we were able to identify a cohort of 137,112 patients with 5,261,732 recorded episodes. Similar to the original study, we randomly selected one episode per patient, with equal probability, to be the index episode, as the intention of the tool is its use at a single time point. Each individual was followed up from the date of their index inpatient or outpatient episode until they either emigrated, died or reached the end of the follow-up period (12 months post-episode). Consistent with the original OxMIS study, we did not exclude individuals who did not reach the end of the follow-up period.

### Outcome definition

By linking the cohort of patients diagnosed with a SMI to the Causes of Death Register, which includes the main and contributory causes of nearly all deaths (>99.7%) [14] recorded in Finland throughout the entire follow-up period, we were able to identify those who had died of suicide, either as the main or contributory causes of death, within 12 months of the index episode. Consistent with the original OxMIS study [15] and research literature [16], we included both certain (ICD-10: X60-X84) and uncertain (ICD-10: Y10-Y34) deaths by suicide.

### Predictors

The OxMIS prediction rule uses 17 predictors representing sociodemographic (i.e., sex, age, educational attainment, benefit receipt), antisocial and suicidal (i.e., previous violent crime, drug use disorder, alcohol use disorder, self-harm), familial (i.e., parental substance use disorders, psychiatric hospitalisation, and suicide), and clinical (i.e., recent treatments of antidepressants or antipsychotics as measured by dispensed (or collected) prescriptions, treatment in inpatient care at the time of assessment, length of stay exceeding 7 days, more than 7 previous episodes, and comorbid depression [in schizophrenia-spectrum disorders]) risk factors for suicide in Sweden. Using the Finnish registries, we created an equivalent set of predictors whose definitions we sought to match as closely as possible (eText). Due to the close geographical proximity between the countries and similarity of their healthcare provision, including the organisation of healthcare and administrative data, nearly all the predictor definitions were similar. There were, however, two differences between the cohort predictors. First, we defined ‘previous violent crime’ as any conviction for an offence ‘against life and health’ (e.g., any assault, manslaughter, and murder) that had occurred prior to the admission. Although this definition is commonly used in Finnish criminological studies [17], it is narrower than the equivalent measure used in the original OxMIS study, which additionally included violent property and sexual crimes, and unlawful threats. Second, the original OxMIS study lacked prescription drug data prior to July 1, 2005, thus necessitating the use of multiple imputation to estimate values for the predictors measuring recent antipsychotic and antidepressant treatment for a large proportion of the participants. In contrast, the Finnish prescription drug data were comprehensive for the duration of the entire follow-up period. There were no missing data in the predictors.

### Analytical approach

Prior to performing any data analyses, we outlined the outcomes, predictors, risk categories, and analytic strategy in a protocol (eText). We then compared the distributions of all predictors across the present Finnish external validation sample with the OxMIS derivation sample. To quantify the associations between the predictors and death by suicide within 12 months of the index episode, we fitted a single logistic regression model to the validation sample. The estimates derived from the model were expressed as adjusted odds ratios (aORs). As equivalent estimates were provided in the original OxMIS study, we then compared the magnitude of associations for each predictor across both samples with Holm-Bonferroni multiple testing correction [18].

Following this, we evaluated the OxMIS prediction model in the Finnish validation sample by calculating the predicted suicide risk one year after the index episode. We did this by calculating the linear predictor (LP; the sum of the products between the untransformed regression coefficients from the original OxMIS study [19] and each of the predictors in the current study) and converting it to the probability scale [P; *P* = 1/(1+exp(-LP)]. This continuous probability score was subsequently used to determine the discriminatory accuracy of the tool, or its ability to differentiate between those who died from suicide and those who did not. We did this by examining the receiver operating characteristic (ROC) curve, which depicts the sensitivity and specificity values of the tool across multiple risk thresholds, and estimating its area under the curve (AUC), where a value of 1 indicated perfect discrimination [20].

In addition to evaluating it as a continuous probability score, we also assessed the performance based on the pre-specified 1% predicted suicide risk cut-off to calculate the number of true and false positives and negatives in our sample and to estimate measures of classification accuracy (e.g., sensitivity, specificity, positive and negative predictive values).

Calibration, or the degree to which predicted risks differ from observed ones, was primarily assessed using a calibration plot. We graphically evaluated the calibration by plotting the observed risks against the predicted risks and visualised their relationship in the form of a smoothed calibration curve, which we estimated using the ‘loess’ algorithm (locally weighted least squares regression smoother) [21].

### Deviations from the protocol

We complemented the graphical evaluation of the model calibration by estimating numeric calibration measures, including the Integrated Calibration Index (ICI), which measures the average absolute difference between the smoothed calibration curve and the observed risks [22]. We also considered the 90^th^ percentile of the absolute difference between observed and predicted risks (E90), as well as the maximum absolute difference between observed and predicted risks (E_max_). For the ICI, E90 and E_max_ measures, a value of 0 indicates that the prediction model is perfectly calibrated [22]. We added balanced accuracy, defined as the mean of the sensitivity and specificity, to the list of classification accuracy metrics [23]. Additionally, we evaluated the performance of OxMIS in accordance with how it has been applied clinically (i.e., in its online calculator), where people with a predicted suicide risk of >5% are assumed to have a predicted suicide risk of 5%.

Stata MP 17 [24] and R 3.5 [25] were used for all analyses. We followed the TRIPOD reporting guidelines for prediction models (eTable 1) [26].

## Results

We identified 137,112 people with an SMI diagnosis in Finland between 1996 and 2017, of whom 101,655 (74%) were diagnosed with a schizophrenia-spectrum disorder and 35,457 (26%) with bipolar disorder. In the 12 months following their index episode, 1475 individuals within this cohort died by suicide, which represents a suicide prevalence rate of 1.1%. In comparison to the Swedish derivation cohort, the Finnish external validation cohort was younger (mean age at assessment: 41 vs. 44 years), had lower rates of previous violent crime (10 vs. 16%), drug use (8 vs. 12%), and self-harm (13 vs. 20%), and recent medication treatments for antipsychotics (45 vs. 54%) and antidepressants (25 vs. 39%), but higher rates of previous alcohol use (19 vs. 15%), benefit receipt (74 vs. 64%) and comorbid depression in schizophrenia-spectrum disorders (43 vs. 32%) (eTable 2). However, there were no clear differences in the association of individual predictors on suicide; the only statistically significant difference was observed for the number of previous episodes exceeding 7 episodes, which was stronger in Finland than in Sweden (aORs: 0.47 vs. 0.77; p_Holm-Bonferroni_ = 0.01; eTable 3).

**Fig. 1:**
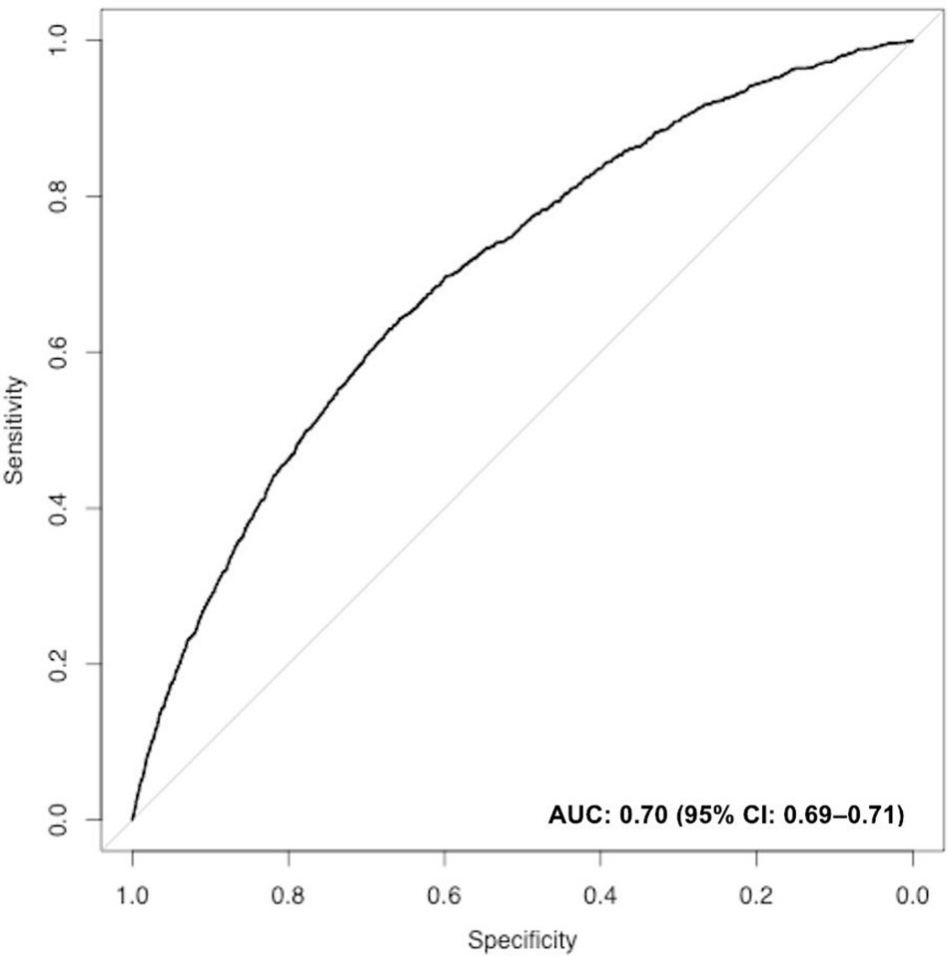
Suicide prediction model discrimination as demonstrated by a receiver operating characteristics curve (ROC; sensitivity against 1-specificity) in the Finnish external validation sample of individuals with severe mental illness (*n* = 137,112). The area under the ROC curve (AUC) is presented with its 95% confidence intervals.

**Fig. 2:**
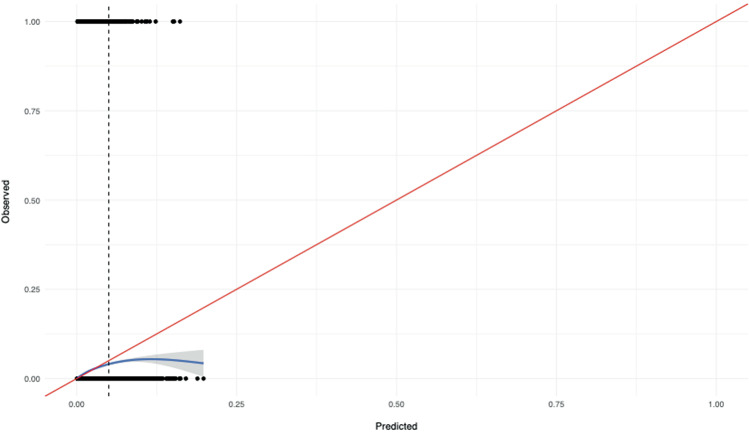
Calibration plot comparing predicted and observed risks of suicide in the Finnish external validation sample of individuals with severe mental illness (*n* = 137,112). Note: The dashed vertical line denotes 5 percent predicted risk, which is the maximum cut-off value presently used in the OxMIS online calculator.

The discriminatory accuracy of the tool was good with an area under the curve of 0.70 (95% CI: 0.69–0.71; Fig. 1). The calibration plot (Fig. 2) indicated that the calibration was very good for those with a predicted probability of 5% or less, which is the maximum risk level used in the OxMIS web calculator at present. However, the plot also showed some overestimation of the tool’s prediction in those with risks >5%, which applied to 1.3% of the cohort (*n* = 1780; eTable 4). Quantitative measures of prediction accuracy confirmed these findings, indicating that the differences between the observed and predicted suicide risks amounted to 0.2% on average (ICI: 0.002) and up to the 90th percentile (E90: 0.002), but the maximum difference was 11.4% (E_max_: 0.114). In complementary sensitivity analyses, we recoded all individuals with a predicted suicide risk of >5% as having a 5% predicted suicide risk, consistent with the way OxMIS online calculator classifies. Using this updated definition, we found that the maximum difference between the observed and predicted suicide risks were reduced from 11.4 to 0.5%, demonstrating very good calibration (ICI: 0.002; E90: 0.002; E_max_: 0.005).

The number of true and false positives and negatives using a pre-specified 1% suicide risk cut-off are presented in eTable 5. Using this cut-off (although cut-offs are not recommended clinically but for research reporting purposes), the tool had a sensitivity of 58.8% (95% CI: 56.3–61.4%), specificity of 70.3% (95% CI: 70.1–70.6%), positive predictive value of 2.1% (95% CI: 2.0–2.3%) and negative predictive value of 99.4% (95% CI: 99.3–99.4%). The balanced accuracy, or the average of the sensitivity and specificity metrics, was 64.6%.

## Discussion

We evaluated the performance of a novel suicide risk assessment tool (OxMIS) in 137,112 people in Finland diagnosed with a schizophrenia-spectrum disorder or bipolar disorder. Good discrimination was indicated by an area under the curve of 0.70. This means that in 70% of the instances when we randomly select two people from our sample, one of whom had died by suicide and the other had not, the tool would give the person who had died by suicide a higher predicted suicide risk score.

Consistent with the original OxMIS study, we discovered that model overestimated risk for extremely high-risk patients (i.e., those with a predicted suicide risk of >5%) and the calibration was poor. However, this only affected a small percentage (1.3%; *n* = 1780) of our sample who had predicted probabilities of this magnitude. In our complementary sensitivity analysis, we observed improved calibration in these patients when we assigned them a suicide risk prediction of no more than 5%. Given our findings, it appears that specific suicide risk predictions above 5 percent are unlikely to be accurate and should therefore be reported as >5%, consistent with how the OxMIS online calculator currently reports risks.

Suicide risk prediction models are commonly limited in the reported performance metrics. Furthermore, a recent systematic review of clinical prediction models in psychiatry found that only 16% of the studies had been validated in wholly independent samples and reported measures of discrimination in development and validation samples [5]. Importantly, the same review found that nearly four out of five (78%) of these studies reported poorer out-of-sample discrimination. In contrast, we found that OxMIS maintained a similar performance from its Swedish validation, with no clear differences between the reported AUCs (range of 0.70–0.71) and balanced accuracy metrics (range of 64.6 and 65.0%).

The strengths of our study included the use of national registers that allowed us to study suicide risk in all individuals with SMI diagnosed in the entire country of Finland over a 22-year period. In contrast to the many validation studies [27], this approach further enabled us to test OxMIS on an external validation sample that was more than twice as large as the derivation sample (*n* = 137,112 vs. *n* = 58,777), thereby providing excellent statistical power. In Finland, the definitions of the predictors were comparable to those in the original study with no missing data. There are two methodological limitations to consider. First, our crime data consisted of criminal conviction records in which assaults, manslaughter, and murder were predefined as violent crimes. In light of this, our definition of ‘previous violent crime’, one of the 17 predictors, was narrower than that of the original OxMIS study, which included violent property and sexual crimes in addition to unlawful threats. Nonetheless, the influence of this misclassification bias remained minimal, as the tool performed similarly in both countries. Second, although we had complete coverage of inpatient care episodes throughout the entire follow-up period, outpatient care data did not begin until 1998, indicating that the prevalence rates for the predictors derived from the patient data were higher for younger cohorts due to left truncation bias. However, despite variations in the birth cohorts included and coverage of outpatient care data, which began in 2001 in Sweden, we obtained similar results to the original OxMIS study.

External validation is only one, albeit key, component of a comprehensive evaluation of any risk prediction model and tool. Other important considerations include the extent to which it is feasible to implement the tool in clinical practice, e.g., the availability of data to calculate risks, transparency in its development and reporting, and for it to gain acceptability among clinicians [28]. Furthermore, any such tool needs linkage to interventions for outcomes to improve. One such intervention in relation to OxMIS is that it underscores safety planning as everyone receives a risk score. At the same time, potential harms need to be considered and OxMIS should be used to support rather than replace clinical decision-making, the latter which will necessarily consider individual, more proximal and contextual factors.

In conclusion, we have conducted a large external validation of one prediction model for suicide (OxMIS). In contrast to the majority of external validation studies in psychiatry, we have pre-specified predictors and outcomes, ensured adequate statistical power (with 1475 outcomes), published a research protocol, and transparently reported our findings, including presenting measures of both discrimination and calibration. We further reduced risks of potential biases by using nationwide registry data with no missing data for the predictors included in the model. With further research on feasibility and work considering how to link risk scores to interventions, OxMIS could assist mental health services in reducing suicide rates in people with SMI.

## Supplementary information

Supplementary tables

Reproducibility checklist

Pre-specified protocol

## Data Availability

Finnish privacy laws prohibit us from making individual-level data publicly available. Researchers who are interested in replicating our work using individual-level data can seek access via Findata (https://findata.fi/en/).

## Code availability

Codes used to generate the results of the present study are available upon request.
