# Supplementary material for: Predicting suicide risk in 137,112 people with severe mental illness in Finland: external validation of the Oxford Mental Illness and Suicide tool (OxMIS)
Source: Transl Psychiatry. 2023 Apr 18;13:126. doi: 10.1038/s41398-023-02422-5 (PMC10113231; doi:10.1038/s41398-023-02422-5)
Supplement: Supplementary file 1 — Supplementary tables [file 41398_2023_2422_MOESM1_ESM.docx]

**eTable 1. TRIPOD Checklist: Prediction Model Validation**

| **Section/Topic** | **Item** | **Checklist Item** | **Page** |
| --- | --- | --- | --- |
| **Title and abstract** | | | |
| Title | 1 | Identify the study as developing and/or validating a multivariable prediction model, the target population, and the outcome to be predicted. | 1 |
| Abstract | 2 | Provide a summary of objectives, study design, setting, participants, sample size, predictors, outcome, statistical analysis, results, and conclusions. | 2 |
| **Introduction** | | | |
| Background and objectives | 3a | Explain the medical context (including whether diagnostic or prognostic) and rationale for developing or validating the multivariable prediction model, including references to existing models. | 3 |
|  | 3b | Specify the objectives, including whether the study describes the development or validation of the model or both. | 3 |
| **Methods** | | | |
| Source of data | 4a | Describe the study design or source of data (e.g., randomized trial, cohort, or registry data), separately for the development and validation data sets, if applicable. | 4 |
|  | 4b | Specify the key study dates, including start of accrual; end of accrual; and, if applicable, end of follow-up. | 4-5 |
| Participants | 5a | Specify key elements of the study setting (e.g., primary care, secondary care, general population) including number and location of centres. | 4 |
|  | 5b | Describe eligibility criteria for participants. | 4 |
|  | 5c | Give details of treatments received, if relevant. | 4 |
| Outcome | 6a | Clearly define the outcome that is predicted by the prediction model, including how and when assessed. | 5 |
|  | 6b | Report any actions to blind assessment of the outcome to be predicted. | N/A |
| Predictors | 7a | Clearly define all predictors used in developing or validating the multivariable prediction model, including how and when they were measured. | 5-6 |
|  | 7b | Report any actions to blind assessment of predictors for the outcome and other predictors. | N/A |
| Sample size | 8 | Explain how the study size was arrived at. | 4-5 |
| Missing data | 9 | Describe how missing data were handled (e.g., complete-case analysis, single imputation, multiple imputation) with details of any imputation method. | 6 |
| Statistical analysis methods | 10c | For validation, describe how the predictions were calculated. | 6-7 |
|  | 10d | Specify all measures used to assess model performance and, if relevant, to compare multiple models. | 6 |
|  | 10e | Describe any model updating (e.g., recalibration) arising from the validation, if done. | 7-8 |
| Risk groups | 11 | Provide details on how risk groups were created, if done. | N/A |
| Development vs. validation | 12 | For validation, identify any differences from the development data in setting, eligibility criteria, outcome, and predictors. | 4,6 |
| **Results** | | | |
| Participants | 13a | Describe the flow of participants through the study, including the number of participants with and without the outcome and, if applicable, a summary of the follow-up time. A diagram may be helpful. | 8 |
|  | 13b | Describe the characteristics of the participants (basic demographics, clinical features, available predictors), including the number of participants with missing data for predictors and outcome. | 8 |
|  | 13c | For validation, show a comparison with the development data of the distribution of important variables (demographics, predictors and outcome). | 8 |
| Model performance | 16 | Report performance measures (with CIs) for the prediction model. | 8-9 |
| Model-updating | 17 | If done, report the results from any model updating (i.e., model specification, model performance). | 9 |
| **Discussion** | | | |
| Limitations | 18 | Discuss any limitations of the study (such as nonrepresentative sample, few events per predictor, missing data). | 10-11 |
| Interpretation | 19a | For validation, discuss the results with reference to performance in the development data, and any other validation data. | 10 |
|  | 19b | Give an overall interpretation of the results, considering objectives, limitations, results from similar studies, and other relevant evidence. | 10 |
| Implications | 20 | Discuss the potential clinical use of the model and implications for future research. | 10 |
| **Other information** | | | |
| Supplementary information | 21 | Provide information about the availability of supplementary resources, such as study protocol, Web calculator, and data sets. | 5 |
| Funding | 22 | Give the source of funding and the role of the funders for the present study. | 12 |

**eTable 2. Baseline characteristics of the Finnish external validation sample compared to the Swedish derivation sample**

|  | **Swedish OxMIS**  **derivation sample** | **Finnish OxMIS**  **validation sample** |
| --- | --- | --- |
| **Predictors** |  |  |
| Total | 58 771 | 137 112 |
|  |  |  |
| Male sex | 29 077 (49%) | 69 319 (51%) |
| Age at the time of assessment (SD) | 44 (13) | 41 (14) |
| Previous violent crime | 9212 (16%) | 13 052 (10%) |
| Previous drug use | 7123 (12%) | 11 224 (8%) |
| Previous alcohol use | 8897 (15%) | 25 296 (19%) |
| Previous self-harm | 11 510 (20%) | 17 680 (13%) |
| Educational level |  |  |
| Primary | 17 814 (35%) | 55 382 (40%) |
| Secondary | 26 449 (52%) | 68 458 (50%) |
| Tertiary | 6489 (13%) | 13 272 (10%) |
| Parental substance use | 5214 (11%) | 14 132 (10%) |
| Parental suicide | 1417 (3%) | 3076 (2%) |
| Diagnosis |  |  |
| Schizophrenia-spectrum disorder (SCZS) | 36 755 (63%) | 101 655 (74%) |
| Bipolar disorder | 22 016 (37%) | 35 457 (26%) |
| Recent treatment |  |  |
| Antipsychotics | 18 401 (54%) | 61 160 (45%) |
| Antidepressants | 13 255 (39%) | 33 983 (25%) |
| Inpatient at the time of assessment | 18 160 (31%) | 40 808 (30%) |
| Length of first inpatient stay >7 days | 24 532 (42%) | 61 915 (45%) |
| Number of previous episodes >7 | 16 686 (28%) | 45 971 (34%) |
| Benefit receipt | 37 210 (64%) | 101 914 (74%) |
| Parental psychiatric hospitalisation | 13 225 (28%) | 36 987 (27%) |
| Comorbid depression | 11 934 (32% of SCZS) | 43 497 (43% of SCZS) |

**eTable 3. Associations between prespecified OxMIS predictors and suicide in the Finnish external validation sample derived from a multivariable logistic regression model as compared to the Swedish derivation sample.**

|  | **Swedish OxMIS**  **derivation sample** | **Finnish OxMIS**  **validation sample** | **P** |
| --- | --- | --- | --- |
| **Predictors** | **aOR [95% CI]** | **aOR [95% CI]** |  |
| Male sex | 1.92 [1.58; 2.33] | 1.90 [1.70; 2.13] | 1.000 |
| Age at the time of assessment (per 10 years) | 0.92 [0.85; 0.99] | 0.87 [0.84; 0.91] | 1.000 |
| Previous violent crime | 0.78 [0.60; 1.02] | 1.11 [0.95; 1.31] | 0.416 |
| Previous drug use | 1.09 [0.84; 1.41] | 1.11 [0.93; 1.32] | 1.000 |
| Previous alcohol use | 1.29 [1.02; 1.63] | 1.14 [1.00; 1.31] | 1.000 |
| Previous self-harm | 2.55 [2.09; 3.11] | 2.33 [2.05; 2.66] | 1.000 |
| Educational level |  |  |  |
| Secondary | 1.24 [1.00; 1.53] | 1.48 [1.32; 1.65] | 1.000 |
| Tertiary | 1.68 [1.24; 2.28] | 1.64 [1.36; 1.99] | 1.000 |
| Parental substance use | 0.70 [0.50; 0.99] | 0.86 [0.71; 1.05] | 1.000 |
| Parental suicide | 1.75 [1.14; 2.69] | 1.72 [1.32; 2.25] | 1.000 |
| Recent treatment |  |  |  |
| Antipsychotics | 1.29 [0.98; 1.69] | 1.05 [0.94; 1.17] | 1.000 |
| Antidepressants | 1.75 [1.29; 2.38] | 1.29 [1.14; 1.45] | 0.966 |
| Inpatient at the time of assessment | 2.95 [2.45; 3.55] | 2.26 [2.01; 2.55] | 0.306 |
| Length of first inpatient stay >7 days | 1.23 [1.00; 1.50] | 1.25 [1.11; 1.40] | 1.000 |
| Number of previous episodes >7 | 0.77 [0.61; 0.97] | 0.47 [0.40; 0.55] | 0.010 |
| Benefit receipt | 0.83 [0.67; 1.02] | 0.83 [0.73; 0.94] | 1.000 |
| Parental psychiatric hospitalisation | 1.20 [0.97; 1.48] | 1.05 [0.92; 1.20] | 1.000 |
| Comorbid depression | 1.27 [1.03; 1.56] | 0.98 [0.88; 1.10] | 0.465 |

*Notes: The p-values were derived from a two-sided test and corrected for multiple testing bias using the Holm-Bonferroni method.*

**eTable 4. The number of observed and predicted deaths by suicide, including their prevalence rates, across categories of predicted risk levels**

| **Predicted risk** | **n** | **Number of suicides** | | **Suicide prevalence** | |
| --- | --- | --- | --- | --- | --- |
|  |  | **Observed** | **Predicted** | **Observed** | **Predicted** |
| 0%-1% | 94 767 | 599 | 472 | 0.6% | 0.5% |
| 1%-2% | 28 323 | 462 | 400 | 1.6% | 1.4% |
| 2%-3% | 7602 | 182 | 183 | 2.4% | 2.4% |
| 3%-4% | 3139 | 95 | 108 | 3.0% | 3.4% |
| 4%-5% | 1502 | 58 | 67 | 3.9% | 4.4% |
| 5%-6% | 703 | 29 | 38 | 4.1% | 5.4% |
| 6%-7% | 380 | 18 | 25 | 4.7% | 6.5% |
| 7%-8% | 241 | 12 | 18 | 5.0% | 7.5% |
| 8%-9% | 168 | 7 | 14 | 4.2% | 8.5% |
| 9%-10% | 122 | 3 | 12 | 2.5% | 9.6% |
| 10% and higher | 165 | 10 | 20 | 6.1% | 12.0% |

**eTable 5. 2x2 table with true and false positives based on a 1% threshold for OxMIS**

|  | **Suicide** | **No suicide** | **Total** |
| --- | --- | --- | --- |
| Elevated risk (>=1%) | 868 | 40 220 | 41 088 |
| Low risk (<1%) | 607 | 95 417 | 96 024 |
| Total | 1475 | 135 637 | 137 112 |
