# Supplementary material for: Predicting suicide risk in 137,112 people with severe mental illness in Finland: external validation of the Oxford Mental Illness and Suicide tool (OxMIS)
Source: Transl Psychiatry. 2023 Apr 18;13:126. doi: 10.1038/s41398-023-02422-5 (PMC10113231; doi:10.1038/s41398-023-02422-5)
Supplement: Supplementary file 3 — Pre-specified protocol [file 41398_2023_2422_MOESM3_ESM.pdf]

Study Title: An external validation study of the Oxford Mental Illness and Suicide (OxMIS) risk assessment tool in Finland

**Principal Investigators:**	Professor Seena Fazel<br>(Department of Psychiatry, University of Oxford)<br><br>Dr Amir Sariaslan<br>(Department of Psychiatry, University of Oxford)
**Co-Investigators:**	Professor Pekka Martikainen<br>(Population Research Unit, Faculty of Social Sciences, University of Helsinki)<br><br>Dr. Thomas R Fanshawe<br>(Nuffield Department of Primary Care Health Sciences, University of Oxford)<br><br>Professor Andrea Cipriani<br>(Department of Psychiatry, University of Oxford)<br><br>Mr. Joonas Pitkänen<br>(Population Research Unit, Faculty of Social Sciences, University of Helsinki)
**Sponsor:**	University of Oxford
**Funding:**	National Institute for Health Research Oxford Health Biomedical Research Centre (BRC-1215-20005).
**Date:**	8 January, 2022

Table of Contents

1. Background and rationale 3

2. Objectives and outcome measures 3

3. Study design 4

4. Participant identification and recruitment 4

4.1. Study participants 4

4.2 Inclusion criteria 4

4.3 Exclusion criteria 4

5. Statistics and analysis 4

5.1 Description of statistical methods 4

5.2 The number of participants 7

6. Data management 7

6.1 Access to data 7

6.2 Data handling and record keeping 7

7. Quality control and quality assurance procedures 8

8. Ethical and regulatory considerations 8

8.1 Declaration of Helsinki 8

8.2. Approvals 9

8.3. Participant confidentiality 9

11. Finance 9

11.1. Funding 9

12. Publication policy 9

References 10

Appendix A 11

Definition of variables 11

1. Background and rationale

The Oxford Mental Illness and Suicide (OxMIS) risk assessment tool was first published in 2019.[1] This prediction model was originally developed in Sweden using population-based registers, and estimates the risk of suicide in patients diagnosed with a severe mental illness (e.g., schizophrenia-spectrum disorders or bipolar disorder). The model was developed using a standard statistical approach (multivariable logistic regression models) and includes 17 pre-specified risk markers based on clinical, socio-demographic, and criminal history variables.

The OxMIS tool generates a probability score of suicide risk within 12 months following discharge from medical care for a psychotic episode, based on 17 predictors. Certain of these risk markers can be imputed as 'unknown' if missing, in which case, the model returns a range of predictive probabilities. OxMIS is intended to support clinical decision-making. It is a significant improvement compared with current suicide risk tools due to the high methodological quality of its development and validation, scalability and ease of use, transparency, and publication of a full range of performance measures (including its discrimination and calibration). Research protocols underlying model development and final coefficients have been published. The model is currently being externally validated in the UK using electronic health record data, and a recent study found it was a feasible approach.[2]

2. Objectives and outcome measures

The objective of the current study is to assess the predictive performance of OxMIS using data from a cohort of patients diagnosed with a severe mental illness in Finland between 1996 and 2017.

The outcome measure, completed suicide within 12 months, will be extracted from the Finnish Causes of Death Register, which includes the main and contributory causes of all deaths recorded in the whole country throughout the time period according to the tenth revision of the International Classification of Diseases (ICD-10). Consistent with the original study[1] and research literature,[3] we will include both certain (ICD-10: X60-X84) and uncertain (ICD-10: Y10-Y34) deaths by suicides.

3. Study design

We will conduct a case-control study of discharged patients with severe mental illnesses and an external validation of the existing OxMIS tool. No new data will be collected as we will use existing routinely collected data from the Finnish nationwide registries.

4. Participant identification and recruitment

4.1. Study participants

Participants will be individuals who were diagnosed with a severe mental illness in Finnish hospitals.

4.2 Inclusion criteria

Residents of Finland who were diagnosed with a severe mental illness between 1 January 1996 and 31 December 2017.

4.3 Exclusion criteria

Individuals who were younger than 15 years or older than 65 years at the time of the discharge.

5. Statistics and analysis

5.1 Description of statistical methods

Performance measures

We will evaluate the calibration and discrimination of the model as recommended in current expert guidelines.[4] Calibration refers to the agreement between the predicted and observed outcomes, while discrimination refers to the ability to distinguish individuals who have died from suicide and those who have not based on a predetermined cut-off (which was set at 1% in the original study). Calibration is usually evaluated graphically through calibration plots, which compare the relationship between observed and predicted outcomes to a diagonal line. Discrimination tests how well a model differentiates different risk categories, and examines true and false positives and negatives. The most

common overall measure of discrimination is the area under the curve (AUC) for specific follow-up times and c index for varying follow-up periods.

Strategy for analysis

We will employ an incremental strategy to assess the prognostic performance of OxMIS in the Finnish cohort.[5,6] First, the individual-level prognostic performance will be evaluated in a simple validation perspective, without any adjustment of the model coefficients for the Finnish population. If the model performs poorly in this new population, it will be updated according to a series of pre-specified steps detailed below.

The following steps will be undertaken, proceeding in each case to the subsequent stage only if diagnostic performance is insufficient. The assessment of prognostic performance between steps (i) to (iv) below will be based on statistical significance (retaining the simplest version model for which the subsequent step provides no statistically significant improvement) and the final assessment of performance based on clinical utility – with a particular focus on the equivalent measures reported in the model development paper.[1]

i. Simple validation: assess the prognostic performance of the existing OxMIS model directly using the Finnish data by applying the existing model directly, without adjusting model coefficients and without adjusting the estimate of baseline risk.

ii. Updating the baseline risk (analogous to the intercept), holding coefficients of predictor variables at their values from the current model. This may also be seen as adjusting for any systematic risk difference that may be attributable to missing covariates (see subsection below).

iii. Updating the baseline risk and performing a simple re-calibration of the coefficients of the predictors. This allows for the inclusion of a single re-calibration parameter, i.e. the effect of predictors on the outcome would be assumed to be the same as in the original study, apart from rescaling.

iv. Additionally re-estimating a subset of the model coefficients. This will be performed only as a last resort as it is likely to lead to a substantively different prediction tool and would allow the associations of the risk markers with the outcome to differ from those found in the original study. If possible, re-estimation will be restricted to a subset of parameters whose coefficients appear to be poorly calibrated.

Cohort definition

The original study was intended to be used at any point in the patient pathway; therefore primary validation will be performed and reported based on a sample of patients who were diagnosed in either inpatient or outpatient care settings in Finland.

Predictors

The current study will examine all 17 predictors included in the OxMIS tool. A list of the variables that will be employed in this analysis is presented below (see appendix A). Given the geographical proximity and similar healthcare service provision, including the ways in which health care and administrative data are organized, between Sweden and Finland, we expect the predictor definitions to be broadly similar across both countries. One potential difference is the definition of previous violent crime convictions. The original study used a somewhat broader measure of violent crime offences than what is currently available in the Finnish database that we have access to. More detailed conviction data has been applied for and is expected to be delivered before 31 December 2022. The updated data will include specific penal codes, which could be used to derive an analogous measure of violent crime as in the original study. The narrower measure of violent crime that is currently available in Finland has nevertheless been used in a wide range of criminological studies to date.[7]

Missing data

We have full coverage of all 17 predictors in the Finnish nationwide registry datasets. This includes the two predictors measuring recent psychotropic dispensed medications (i.e., antipsychotics and antidepressants), which had to be partly imputed in the original study due to the fact that the Swedish Prescription Drug Register started on 1 July 2005 whilst the study started on 1 January 2001. The Finnish Prescription Drug Register, however, started on 1 January 1995, thus covering the entire follow-up period of the current study.

Censoring

Similar to the original study, we intend to ignore right-censoring (e.g., emigration and non-suicide deaths) during the follow-up period as it is relatively short and the number of censored individuals are likely quite small. It also reduces complexity of models.

Predicted probabilities

Results will be primarily presented based on risk probabilities, but predictive performance will also be assessed using contingency tables and summary statistics.

Presentation of results

Findings will be presented using both calibration and discrimination, graphically and using tables and summary statistics as appropriate. These will include calibration plots; sensitivity and specificity; AUC.

The report will include descriptive information about the cohort and how it was defined, including any differences from the original cohort in definitions of variables. As far as possible we will follow published guidance on reporting validation study results.[8–10]

5.2 The number of participants

Preliminary data suggest a total sample size exceeding 137,000 individuals, nearly twice that of the original study (n=75,158). A simulation study recommended ballpark minimum figures of 100 events for validating a clinical prediction rule derived using a logistic regression model.[11] If we were to identify an equivalent baseline one-year suicide risk rate as in the original study (0.8%), we would thus have nearly 11 times as many outcome events (i.e. suicides) as the minimum recommendation for validation studies (1096 vs. 100).

6. Data management

6.1 Access to data

Direct access will be granted to authorised representatives from the University of Oxford and the host institution, University of Helsinki, for monitoring and/or audit of the study to ensure compliance with regulations.

6.2 Data handling and record keeping

The raw pseudonymized data (i.e., using encrypted personal identification numbers) is currently available on the servers of Statistics Finland, which one representative of the University of Oxford

has access to via the high-security remote desktop service of the agency. To gain entry to the remote desktop server ("FIONA"), a user needs to first log in to an accredited Finnish university or governmental agency using a Virtual Private Network (VPN) connection. Once this has been accomplished, the user can then connect to FIONA via a two-step authentication process (e.g., inputting a username and password in addition to accepting the connection on a mobile phone with a number that has been pre-registered by Statistics Finland). It is not possible for FIONA users to transfer any files, either to or from the system. Output files may be exported to the e-mail address of the user by staff members of Statistics Finland following an inspection to determine that they do not include any sensitive individual-level data.

A university researcher at the University of Oxford will initially merge and clean the raw data according to tidy data principles,[12] and subsequently fit the models and generate the proposed post-estimation statistics for model fit. The entire process will be documented using Stata do-files and the RMarkdown reporting tool[13] to facilitate future replication efforts. All data files and codes necessary to replicate the analyses will be stored on the FIONA platform.

7. Quality control and quality assurance procedures

The study will be conducted in accordance with the current approved protocol, relevant regulations, and standard operating procedures.

8. Ethical and regulatory considerations

8.1 Declaration of Helsinki

The Investigators will ensure that this study is conducted in accordance with the principles of the Declaration of Helsinki.

NB. The 2008 Declaration of Helsinki provides detail on what must be included in a protocol: funding, sponsorship, affiliations and potential conflicts of interest, incentives to participate and compensation for harm.

8.2. Approvals

This project has been approved by the Ethics Board of Statistics Finland (TK-53-1490-18) and the Finnish Institute for Health and Welfare (THL/2180/14.02.00/2020).

The Investigator will submit and, where necessary, obtain approval from the above parties for all substantial amendments to the original approved documents.

8.3. Participant confidentiality

The data provided to the Oxford researcher by Statistics Finland will be fully pseudonymized. The participants are identified by an encrypted participant ID on the electronic database. All documents will be stored securely and only accessible by study staff and authorised personnel. The study fully complies with Finnish privacy laws.

11. Finance

11.1. Funding

No specific financial arrangements have been made for this study. The project sits within Oxford Health's BRC strategy.

12. Publication policy

The Investigators will be involved in reviewing drafts of the manuscripts, abstracts, press releases and any other publications arising from the study. Authorship will be determined in accordance with the ICMJE guidelines and other contributors will be acknowledged.

References

1. Fazel S, Wolf A, Larsson H, Mallett S, Fanshawe TR. The prediction of suicide in severe mental illness: development and validation of a clinical prediction rule (OxMIS). *Transl Psychiatry*. 2019;9(1):98.

2. Senior M, Burghart M, Yu R, et al. Identifying Predictors of Suicide in Severe Mental Illness: A Feasibility Study of a Clinical Prediction Rule (Oxford Mental Illness and Suicide Tool or OxMIS). *Front Psychiatry*. 2020;11:268.

3. Neeleman J, Wessely S. Changes in classification of suicide in England and Wales: time trends and associations with coroners' professional backgrounds. *Psychol Med*. 1997;27(2):467-472.

4. Collins GS, Ma J, Gerry S, et al. Risk Prediction Models in Perioperative Medicine: Methodological Considerations. *Curr Anesthesiol Rep*. 2016;6(3):267-275.

5. Steyerberg EW. *Clinical Prediction Models*. Springer; 2019.

6. Su TL, Jaki T, Hickey GL, Buchan I, Sperrin M. A review of statistical updating methods for clinical prediction models. *Stat Methods Med Res*. 2018;27(1):185-197.

7. Ramakers A, Aaltonen M, Martikainen P. A closer look at labour market status and crime among a general population sample of young men and women. *Adv Life Course Res*. 2020;43:100322.

8. Bouwmeester W, Zuithoff NPA, Mallett S, et al. Reporting and Methods in Clinical Prediction Research: A Systematic Review. *PLOS Med*. 2012;9(5):e1001221. doi:10.1371/journal.pmed.1001221

9. Collins GS, de Groot JA, Dutton S, et al. External validation of multivariable prediction models: a systematic review of methodological conduct and reporting. *BMC Med Res Methodol*. 2014;14(1):40. doi:10.1186/1471-2288-14-40

10. Collins GS, Reitsma JB, Altman DG, Moons KG. Transparent reporting of a multivariable prediction model for individual prognosis or diagnosis (TRIPOD): the TRIPOD Statement. *BMC Med*. 2015;13(1):1-10.

11. Vergouwe Y, Steyerberg EW, Eijkemans MJC, Habbema JDF. Substantial effective sample sizes were required for external validation studies of predictive logistic regression models. *J Clin Epidemiol*. 2005;58(5):475-483.

12. Wickham H. Tidy Data. *J Stat Softw*. 2014;59:1-23.

13. Xie Y, Allaire JJ, Grolemund G. *R Markdown: The Definitive Guide*. CRC Press; 2018.

Appendix A

Definition of variables

The table refers to the variables as defined in the original Swedish cohort.

#	Variable	Type	Notes
1	Sex	Binary	Male/female
2	Age	Continuous	Age at assessment (allowed range: 15-65). The measure is divided by a constant (10 years) to aid with the interpretation of the regression model.
3	Previous violent crime	Binary	Previous conviction for a violent offence, defined as homicide, assault, robbery, arson, any sexual offense (rape, sexual coercion, child molestation, indecent exposure, or sexual harassment), illegal threats, or intimidation.
4	Previous drug use	Binary	Previous diagnosis of drug use disorder.
5	Previous alcohol use	Binary	Previous diagnosis of alcohol use disorder.
6	Previous self-harm	Binary	Previous diagnosis of alcohol use disorder.
7	Highest education level	Categorical	Formal schooling: secondary (to age 16), upper secondary (to age 18), post-secondary (past 18) at the time of assessment.
8	Parental drug or alcohol use	Binary	Parental diagnosis of drug or alcohol use disorder.
9	Parental suicide	Binary	Parental death by suicide.
10	Recent treatment - antipsychotic	Binary	Prescribed antipsychotics (ATC

			code N05A [except N05AN01]) at least once up to 6 months prior to the assessment.
11	Recent treatment - antidepressant	Binary	Prescribed antidepressants (ATC code N06A) at least once up to 6 months prior to the assessment.
12	Inpatient at the time of assessment	Binary	Discharged from an inpatient care setting.
13	Length of first inpatient stay > 7	Binary	Hospitalized for at least one week during their first inpatient stay.
14	Number of previous episodes > 7	Binary	Previous patient episodes for schizophrenia-spectrum or bipolar disorders. Excluding current episode. A patient episode is defined as one outpatient specialist review (in a mental health service) or one hospitalization (including an overnight stay); two hospitalizations would be considered two periods of care.
15	Benefit receipt	Binary	Receipt of disability pension and/or social assistance benefits prior to the assessment.
16	Parental psychiatric hospitalization	Binary	Parental hospitalization for a psychiatric disorder.
17	Comorbid depression	Binary	Comorbid depression diagnosis, only in those with schizophrenia-spectrum disorders.
